# Impact of the *in situ* rise in hydrogen partial pressure on graphene shape evolution during CVD growth of graphene†
†Electronic supplementary information (ESI) available. See DOI: 10.1039/c7ra13169k


**DOI:** 10.1039/c7ra13169k

**Published:** 2018-02-21

**Authors:** Zewdu M. Gebeyehu, Aloïs Arrighi, Marius V. Costache, Clivia M. Sotomayor-Torres, Maria J. Esplandiu, Sergio O. Valenzuela

**Affiliations:** a Catalan Institute of Nanoscience and Nanotechnology (ICN2), CSIC, The Barcelona Institute of Science and Technology (BIST), Campus UAB, Bellaterra, 08193 Barcelona, Spain. Email: zewdu.messele@icn2.cat; Email: mariajose.esplandiu@icn2.cat; b Universitat Autònoma de Barcelona (UAB), Bellaterra, E-08193, Spain; c Institució Catalana de Recerca i Estudis Avançats (ICREA), 0870 Barcelona, Spain. Email: SOV@icrea.cat

## Abstract

Exposing graphene to a hydrogen post-etching process yields dendritic graphene shapes. Here, we demonstrate that similar dendritic structures can be achieved at long growth times without adding hydrogen externally. These shapes are not a result of a surface diffusion controlled growth but of the competing backward reaction (etching), which dominates the growth dynamics at long times due to an *in situ* rise in the hydrogen partial pressure. We have performed a systematic study on the growth of graphene as a function of time to identify the onset and gradual evolution of graphene shapes caused by etching and then demonstrated that the etching can be stopped by reducing the flow of hydrogen from the feed. In addition, we have found that the etching rate due to the *in situ* rise in hydrogen is strongly dependent on the confinement (geometrical confinement) of copper foil. Highly etched graphene with dendritic shapes was observed in unconfined copper foil regions while no etching was found in graphene grown in a confined reaction region. This highlights the effect of the dynamic reactant distribution in activating the *in situ* etching process during growth, which needs to be counteracted or controlled for large scale growth.

## Abstract 2

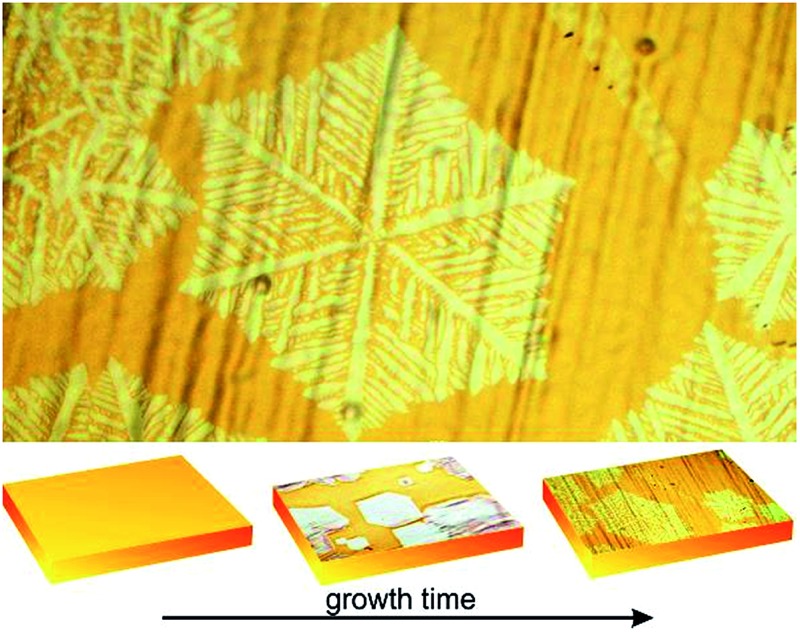
Growth to etching transformation following *in situ* rise in hydrogen with time results in dendritic graphene.

## Introduction

Graphene, a two-dimensional (2D) single-atom-thick crystal of sp^2^-bonded carbon, has attracted great attention because of its outstanding physical properties.^1^–^3^ Its large carrier mobility, low-spin–orbit interaction and carrier-density tunability make it very attractive for electronic and spintronic applications.^1^–^4^ Such applications require the production of graphene on large scales with extremely high reproducibility and large grains size. Among the known methods, chemical vapor deposition (CVD) on the surface of metal catalysts such as copper and nickel appears as the most promising approach.^5^–^9^

Despite continuous progress, CVD grown graphene, covering wafer size areas, is typically polycrystalline comprising small grain sizes which cause deterioration of the physical and chemical properties. To avoid the grain boundary created by merging of different domains, considerable efforts have been made to grow single-crystal isolated graphene flakes. However, isolated graphene flakes can adopt a large variety of shapes, including compact and dendritic structures, which are determined by the growth pressure,^10^ the hydrogen/methane (H_2_/CH_4_) ratio^11^,^12^ and the growth temperature.^13^,^14^ Such intrinsic complexity of CVD growth leads to a large spread of experimental results, even when using similar nominal growth conditions. In particular, dendritic structures have been reported by several groups in the low CH_4_ flow rate regime.^14^–^16^ The microscopic mechanism that leads to these structures is a subject of debate. They were ascribed to the kinetics of carbon flux attachment to the carbon edges, the anisotropic surface diffusion due to the underlying crystallinity^14^ or to the degree of roughness^15^ of the copper foil and to a surface diffusion controlled growth mechanism arising from the limited amount of carbon source.^16^ Remarkably, similar dendritic shapes were achieved by etching the CVD grown graphene after growth (post-etching).^17^,^18^ In those experiments, graphene is first grown following a standard procedure using a CH_4_ in Ar/H_2_ gas mixture, then the CH_4_ is switched off and, finally, graphene is etched with Ar/H_2_. In other reports, gaseous oxidants were considered to be the main responsible for graphene etching, giving place to lines and holes with hexagonal shapes.^19^

All of the above reports, which focus primarily on either growth or etching of graphene, are seemingly disconnected. However, CVD growth of graphene actually involves a competition between growth and etching.^20^ It is thus possible that the dendritic shapes might not be only the result of a surface diffusion controlled growth mechanism but also of an etching takeover over time without modification of growth conditions. Therefore, understanding the origin of the dendritic shapes is critical to gain insight into the dynamic processes involving growth and etching during deposition, which has often been ignored, and can lead to novel approaches to further develop CVD growth.

Here we demonstrate that dendritic shapes can be obtained within a given growth process promoted by the etching takeover at long growth times. We have studied systematically the shape evolution the graphene flakes at shorter times under the same growth conditions. We have found that, indeed, after an initial growth, the competing backward etching starts dominating, shaping the graphene into dendritic structures. We have ascribed the etching and formation of dendrites to an *in situ* rise in H_2_ partial pressure during growth. We also show that the confinement of the copper foil impacts on the growth/etching balance. Etching is selectively observed in unconfined regions. This is a very important finding since it can be an additional source of heterogeneity that must be taken into consideration for explaining the wide dispersion of results among research groups. In addition, by tuning the amount of H_2_ after a standard growth period (before etching starts to dominate), we demonstrate that oxidative etching coexists with the reductive etching mediated by the *in situ* rise in H_2_ partial pressure. The previously observed oxidative etching^19^ becomes evident at low H_2_ flow rates and is characterized by the formation of hexagonal holes carved into the graphene flakes.

## Experimental

### Growth of CVD graphene

Graphene growth was performed under reduced pressure on copper foil using a 1 inch quartz tube reactor. Briefly, copper foil was annealed in Ar/H_2_ (450/50 sccm) for 1 hour at 1000 °C. The copper substrate was exposed to 1 sccm O_2_ flow during 2 min, while keeping the same Ar flow and H_2_ switched off, in order to decrease graphene nucleation density by passivating catalyst active sites. Growth was then performed in Ar/H_2_/CH_4_ (450/50/1 sccm) for various growth times at 1000 °C, after which the samples were fast-cooled down in Ar/H_2_ (450/50 sccm) atmosphere by taking the tube out of the furnace. Unless specified, these are the standard growth conditions selected throughout the paper. The growth was systematically studied with specific configurations of the copper substrate in order to analyze its influence on the concentration of reactants. To demonstrate the *in situ* increase of H_2_, and the transition to oxidative etching, we performed a series of experiments in which the H_2_ flow rate was reduced during an additional 10 min growth period. For further details, refer to the ESI.† The quality of graphene grown on the copper film by CVD was verified by an optical microscope, scanning (SEM) and transmission (TEM) electron microscopes.

### Characterization

Optical microscope (Nikon Eclipse LV100 ND) characterization was done on as-grown graphene on copper foil. After growth, the graphene/copper substrate was heated upto 180 °C on a hot plate for 2 min for better optical visualization. The graphene-free copper surface changes color due to oxidation at such temperature and the graphene-covered copper remains unchanged which helps to identify graphene flakes by color contrast difference.

Scanning electron microscopy SEM (SEM Magellan 400L XHR) operated at an acceleration voltage of 10 kV was used to characterize the morphology and shape evolution of graphene domain on copper foil. The crystalline structure of graphene was characterized with high resolution transmission electron microscopy (HRTEM FEi Tecnai G2 F20) operated at 200 kV after transferring the as-grown graphene to a TEM grid (see ESI for transfer techniques†).

## Results and discussions

### Investigation of growth mechanism

**Fig. 1:**
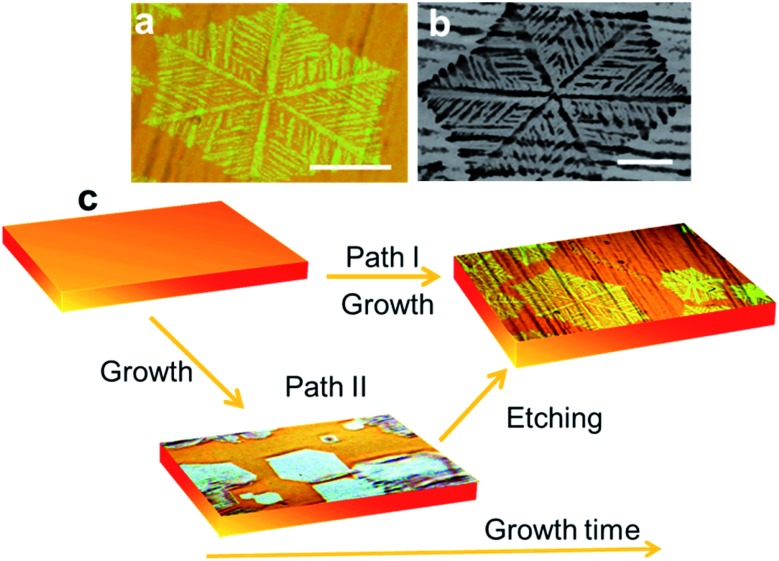
(a) Optical and (b) SEM picture of as-grown graphene on a copper foil. (c) Scheme showing the two possible pathways leading to the final graphene structure. In prior work, direct growth of dendritic shapes was reported (Path I). We propose that the same structures can be the result of a two-step mechanism (Path II) consisting of growth and subsequent etching. The scale bars are 50 μm.

Fig. 1a and b show optical microscope and scanning electron microscopy images of as-grown graphene on copper foil, respectively. The growth time for these flakes was 60 min under the standard conditions specified before. As seen in Fig. 1a and b, the graphene domains have a dendritic structure and cover an area with a hexagonal shape. These results are similar to those in previous reports, which were ascribed to an anisotropic growth.^14^ The flakes are also similar to those reported in post-growth hydrogen-assisted etching.^17^,^18^ A comparison of these reports, in terms of morphology, size of the flakes and main processes is tabulated in Table 1.

**Table 1:** Comparison of reports on dendritic structures: dendritic flakes from surface-diffusion limited growth process (ref. 14, 15 and 16), from hydrogen post-etching (ref. 17) and from growth to etching transformation due to an *in situ* rise in H_2_ at longer growth time (this report)

Morphology	Mechanism and process conditions	Size of flakes	Process time	Reference
Flakes with dendritic edge	Growth with controlled CH_4_/H_2_ ratio	Not specified	Not specified	14
Flakes with dendritic edge	Growth with controlled CH_4_/H_2_ ratio	70 μm	15 min growth	15
Flakes with dendritic edge	Growth at low CH_4_/H_2_ ratio	100 μm	30 min growth	16
Flakes with etched edge	H_2_ post-etching (fractal etching) after growth	Not specified	30 min growth and 5 min post-etching	17
Flakes with dendritic edge	Growth to etching transformation due to *in situ* rise in H_2_ with time	Few hundred microns	20 min exclusive growth followed by time dependent etching	This report

As illustrated in Fig. 1c, we propose two possible paths for the resulting dendritic graphene shape: growth or growth followed by etching. That is, the flake morphology could be (i) the outcome of surface-diffusion limited growth (growth outcome) as in previous reports^14^,^15^ (Fig. 1c Path I) or (ii) the result of a competing etching process that dominates after a certain growth time under the same growth condition (Fig. 1c Path II).

It is, therefore, crucial to discriminate these two mechanisms in order to gather better understanding of the CVD graphene growth process. For this purpose, we performed a time-dependent growth experiment.

### Time-dependent graphene shape evolution

**Fig. 2:**
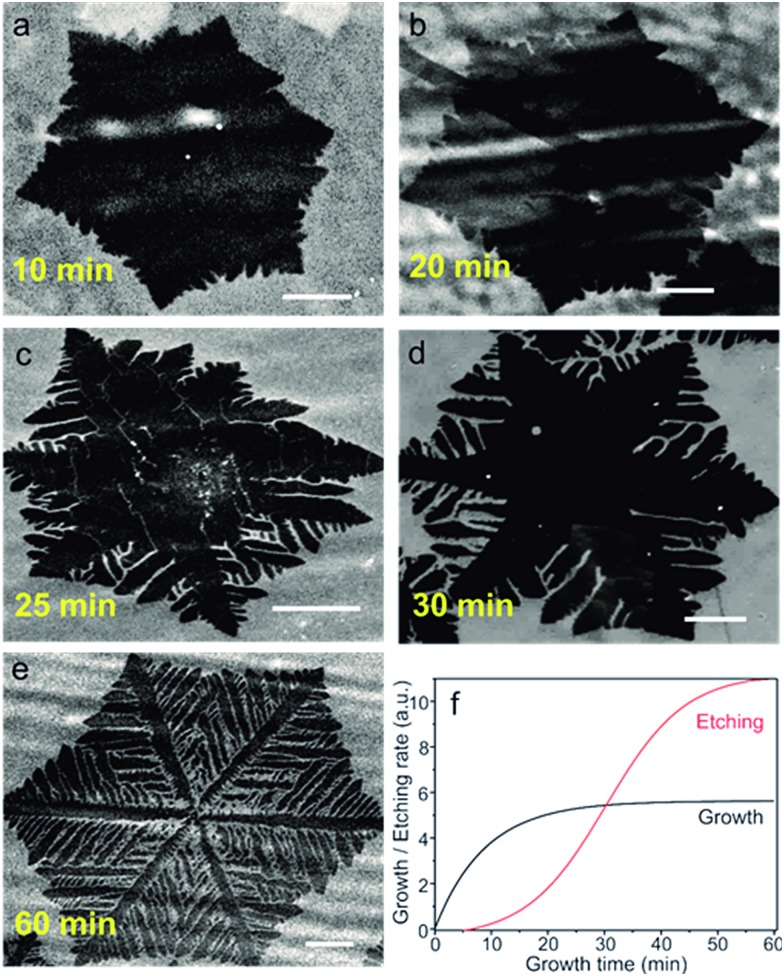
SEM images depicting the evolution of the graphene flake shapes (darker region for graphene) at different growth times (a) 10 min (b) 20 min, (c) 25 min, (d) 30 min, (e) 60 min. The scale bars are 25 μm. (f) Qualitative diagram showing the growth (black line) and etching (red line) profile, which explains the results in (a)–(d). At short times (<15 min), the flakes grow rapidly, as etching process is slow. As the etching time increases, the growth slows down. The dendritic structures appear at times >20 min when the etching takes over.

In order to verify if Path II is possible, we vary the growth times to determine the evolution of the graphene domain shape. Fig. 2a–e show a series of SEM images of typical individual flakes on copper foil for growth times of 10, 20, 25, 30 and 60 min. The size and shape distributions of graphene are highly reproducible for independent growths experiments. We clearly observe that the flakes grow rapidly without dendritic shape for times below 10 min (Fig. 2a and S1a†). Between 10 and 20 min, the growth slows down significantly. At 20 min, the size of the flakes barely increases (Fig. 2b). Remarkably, at 25 min and longer times, a dendritic shaping of the flakes becomes evident, which indicates a progressive takeover of an etching process (Fig. 2c to e and S1b†).

The growth/etching competition is qualitatively represented in Fig. 2f, where a growth zone and an etching zone are identified. In the initially growth zone, up to about 10 min, etching must be negligible, leading to a fast increase of the graphene domain size. However, at times beyond 10 min, the etching becomes significant enough to counteract the growth, as demonstrated by the small overall increase of the domain size. When the growth time exceeds 20 min, the etching takes over and the flakes start changing to dendritic shapes.

As advanced previously, our growth results (growth followed by etching) have similarities with previous studies in which graphene was carved by post-etching with the addition of external doses of hydrogen.^17^,^18^ As a consequence, the gradual increase of the etching process with the resulting dendritic shape of the graphene domains suggests that the H_2_ partial pressure exceeds the required amount for growth and the CVD dynamics is shifted to etching. Since the H_2_ flows from the feed is constant, a non-negligible amount of H_2_ must accumulate in the reaction path during growth. We ascribe this observation to an *in situ* rise in hydrogen partial pressure resulting from the decomposition of methane and the difficulty to pump-out the light hydrogen molecules from the furnace. Indications of the local increase of H_2_ with growth time have been also observed in a recent paper.^22^ In our experiment, the excess H_2_ enhances graphene etching from the edges: because of the functional groups and dangling bonds, the chemical reactivity of graphene edge carbon atoms is higher than the perfectly bonded carbon atoms in the basal plane.^23^

### Systematic control of hydrogen etching

**Fig. 3:**
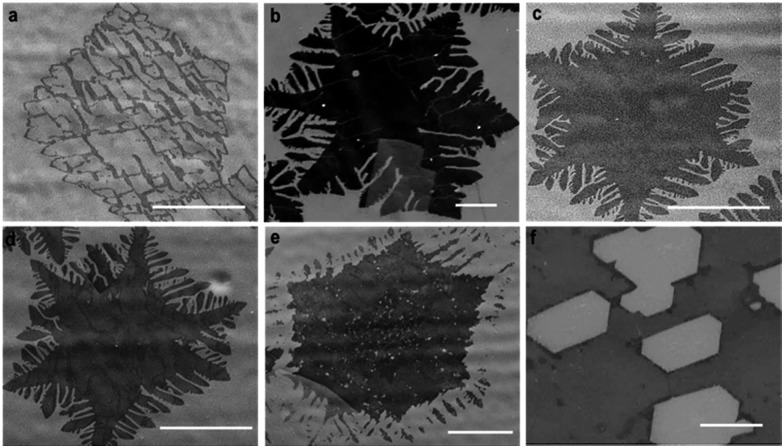
Etching profiles for 20 min with the standard growth method followed by additional 10 min growth with reduced H_2_ flows. The SEM images (darker region for graphene) depict the transition from high to low hydrogen flow. (a) 60 sccm: graphene shape is strongly etched at high hydrogen flows. (b) 50 (c) 30 and (d) 20 sccm: dendritic etching rate decreases gradually in the sequence, (e) 10 sccm: hole etching starts to be observed, (f) 5 sccm purely oxidative etching. The scale bars are (b)–(e) = 25 μm and (f) = 10 μm.

We performed a series of experiments in the etching zone in which we reduced the external flow rate of H_2_. The purpose is to compensate for the *in situ* rise in the H_2_ partial pressure during growth and thus reduce the etching that is induced by it. In each of our experiments, we first grew graphene during 20 min with our standard parameters, and then we decreased the H_2_ flow rates from 50 sccm down to 30, 20, 10, 5, and 0 sccm for additional 10 min keeping the other parameters constant (Ar 450 sccm, CH_4_ 1 sccm). For further comparison, we performed an additional experiment in which the H_2_ flow rate was increased to 60 sccm. Fig. 3a to f show typical SEM images of the resulting graphene flakes. These images should be compared with those in Fig. 2d where the H_2_ flow rate was kept constant for 30 min. For 60 sccm, we observe that the etching process is very aggressive leading to an almost complete disappearance of the flakes (Fig. 3a). The etching appears to carve holes as in the case of high H_2_ flow in ref. 17. As the H_2_ flow decreases, we observe dendritic etching, which then decreases gradually as the flow rate of H_2_ decreases further (Fig. 3b–e). These findings demonstrate that the dendritic-like etching is driven by an *in situ* rise in H_2_.

At 10 sccm the etching process exhibits a combination of hexagonal hole-led and dendritic-type etching (Fig. 3e), which suggests a transition in the etching mechanism. Notably, for a flow rate of 5 sccm, dendritic etching is no longer present. In contrast, the graphene flakes exhibit hexagonal holes (Fig. 3f and S2a†) that are reminiscent of the etching in an oxidant environment.^18^

We interpret the transition to oxidative etching as follows. Besides acting as an activator of the surface-bound carbon and as an etching reagent, H_2_ plays a role for neutralizing oxygen based species, which could originate either from oxygen-based impurities in the feedstock gases or be already present in the furnace.^18^ At low H_2_ flows, the concentration of H_2_ is not high enough to fulfill such a role, triggering the oxidative etching mechanism. At 0 sccm the etching is so aggressive that the graphene flakes are completely removed from the copper foil *via* hole-led etching (Fig. S2b†).

Interestingly, and in line with previous studies,^18^ the oxidative etching yields anisotropic hexagonal etched patterns that are aligned with each other, which can be used as evidence that the grown graphene is a single crystalline structure.^24^,^25^ The single crystal structure of graphene domains was further confirmed by selected area electron diffraction (SAED), with a high-resolution TEM (HRTEM) (Fig. S3†). Additionally, we have performed Raman spectroscopy (Horiba T64000 Raman spectrometer, 532 nm laser) for a typical graphene flake, before the etching takeover, at three different locations. The Raman spectra presented practically indistinguishable profiles, indicating a high degree of homogeneity (see ESI, Fig S4a and b†).

### Effect of confinement of the copper foil on the induced etching by the *in situ* rise in hydrogen

**Fig. 4:**
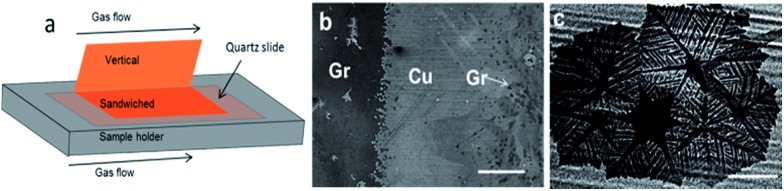
(a) Schematics showing the geometrical configuration of the copper foil during typical growth process (bent copper foil), (b) SEM image near the bent region containing both vertical (right) and sandwiched parts (left) and (c) zoomed image of typical graphene islands in the unconfined copper foil. The image in (c) shows the dendritic shape structure due to etching caused by the *in situ* rise in hydrogen partial pressure. The scale bars are (b) 500 μm and (c) 100 μm.

The confinement of the copper foil is known to play a significant role in determining the local partial pressure of gasses species.^21^,^26^,^27^ It is, therefore, likely that confinement also impacts the growth/etching competition process. Considering this, we examined the inhomogeneities of the *in situ* H_2_ rise in different regions of the copper foil by observing the local graphene etching level. We bent the copper foil into two parts to make half part vertical (unconfined) and half part horizontal (confined). The horizontal part was sandwiched between a quartz glass slide and the quartz substrate holder (Fig. 4a, see also ESI for methods and Fig. S5†). Due to the difference in confinement and exposure to the gas flow, we expect differences in the etching process during the 60 min growth time. Fig. 4b shows a large area SEM image that covers both vertical and sandwiched regions. Even in such large scale, it is readily observed that the growth is very different. The darker region (left) corresponds to sandwiched-horizontal portion and is fully covered with graphene. The brighter region (right) corresponds to the vertical copper foil and only presents small islands of graphene (observed as dark spots). We have also found that the growth morphology of graphene differs in the two regions. In the confined region graphene is featureless. In contrast, by zooming into the unconfined region, we find that graphene flakes are etched into dendritic shapes (Fig. 4c).

In the confined region, the gas flow velocity is much slower due to the reduced space between the copper foil surface and the sample holder, resulting in a thicker boundary layer where mass transport of carbon and H_2_ to the copper foil is slow.^21^ Consequently, the growth rate and its accompanying H_2_ production become slower. However, on the unconfined region the boundary layer is thinner. There, the mass transport of the reactants to the copper foil is fast, facing a higher concentration of carbon promoting growth^21^ and a higher flow of H_2_. The H_2_ enrichment at the catalyst surface plus the local rise in H_2_ produced from the reaction as a byproduct favors the etching reaction. Consequently the etching process ends up dominating over the growth leading to dendritic graphene shape. Hence, these findings indicate that etching can be suppressed by subjecting the catalyst in a confined reaction region.

## Conclusions

We have demonstrated that the graphene morphology changes from a compact to a dendritic structure over growth time without modifying the external growth conditions. The dendritic shape is a consequence of a competing backward etching reaction, which starts dominating over the growth at long times due to an *in situ* hydrogen concentration increase. This contrasts with previous reports, which ascribed such dendritic structures to a surface diffusion controlled growth. The results are further confirmed by decreasing the external flow of hydrogen, which leads to a suppression of the etching. We have found however, that further growth at low hydrogen flows is slowed down due to the emergence of oxidizing etching, which indicates that the concentration of gaseous oxidants also increases. Moreover, the etching induced by the *in situ* increase of hydrogen concentration is also very dependent on the copper foil geometrical arrangement, yielding highly etched graphene on unconfined copper foil regions. These findings demonstrate that local fluctuations of the reactants during growth have a large impact on the resulting graphene morphology. They provide information on the etching process of graphene and highlight the critical importance of confinement aspects and the balance of gases as a source of heterogeneities, which can account for the wide dispersion of results among research groups. Overall, they also suggest that a careful balance of the local concentration of all reagents at any given time of the growth process is necessary to optimize the growth of graphene.

## Conflicts of interest

There are no conflicts to declare.

## Supplementary Material

Supplementary information
