# Supplementary material for: Impact of the in situ rise in hydrogen partial pressure on graphene shape evolution during CVD growth of graphene
Source: RSC Adv. 2018 Feb 21;8(15):8234–9. doi: 10.1039/c7ra13169k (PMC5830861; doi:10.1039/c7ra13169k)
Supplement: RA-008-C7RA13169K-s001 [file RA-008-C7RA13169K-s001.pdf]

Electronic Supplementary Material (ESI) for RSC Advances.
This journal is © The Royal Society of Chemistry 2018

Supporting information

Impact of the *in-situ* rise of hydrogen partial pressure on graphene shape evolution during CVD growth of graphene

Zewdu M.Gebeyehu[a,b*], Aloïs Arrighi[a,b], Marius V. Costache[a], Clivia M. Sotomayor-Torres[a,c], Maria J. Esplandiu[a,b*], Sergio O. Valenzuela[a,c*]

Methods

Copper foil preparation

A 25 μm thick copper foil (99.8%, Alfa Aesar, stock #13382) was cleaned in acetone and isopropyl alcohol (IPA) for 10 min each to remove organic contaminants. In order to remove surface oxides and other inorganic contaminants, the copper foil was chemically etched using acetic acid/water (1/3) and then thoroughly rinsed with DI water followed by blow-drying with nitrogen gas. For the inspection of the hydrogen etching impact on the catalyst geometrical arrangement (confinment), the copper foil was subjected to a different confinement. In brief, the copper foil was bent into two parts to make half of the copper foil part vertical (non-confined) and half part horizontal (confined) by sandwiching (Fig. 5)it between the sample holder and the quartz glass slide.

Growth of graphene

Growth was performed using a (Lindberg/Blue M, Asheville, NC, USA) furnace and 1-meter long 1-inch diameter quartz tube. Cleaned copper foils were loaded into the growth chamber and the tube was evacuated and all the gases were purged for 5 min in vacuum before heating up. The copper foil was annealed Ar (99.9992% purity) 450 sccm and H_2 (99.999% purity) 50 sccm at 1000 ^{0}C. O_2 was flowed with 1 sccm for 2 min with Ar (450 sccm) and 0 sccm of hydrogen for oxidative passivation of the copper foil just right before the growth started. Then graphene was grown in Ar 450 sccm, H_2 50 sccm and CH_4 1 sccm (99.995 %) at 1000 ^{0}C. After growth, the CH_4 was turned-off and the system was fast cooled down to room temperature under Ar/H_2 atmosphere by taking the quartz tube out of the furnace. As-grown graphene on copper foil was heated on a hot plate for 2 min to increase the contrast difference between graphene and copper during microscopic imaging.

Transfer of graphene to SiO_2/Si and TEM grid

The graphene flakes were transferred onto **SiO_2/Si and** TEM grid with polymethyl methacrylate (PMMA) assisted transfer for TEM and Raman characterization. PMMA (A4 950 K) was spin coated on Copper/graphene layer at 3000 rpm for 40 seconds (3x)followed by air drying overnight. The underneath graphene was removed floating on 20% nitric acid for 2.5 min followed by rinsing with deionized water. Then the copper was etched floating on a 0.5 M $FeCl_3$ solution. Then PMMA/graphene stack was cleaned with deionized water (3x), 10 %

HCl for 10 min and then again with deionized water (2x). The PMMA/graphene was fished out from deionized water using **SiO_2/Si (440 nm) or** TEM grid , dried and cured at 85 ^{0}C for 30 min. PMMA was removed by soaking the PMMA/graphene stack in hot acetone at 50 ^{0}C and rinsed with isopropanol alcohol

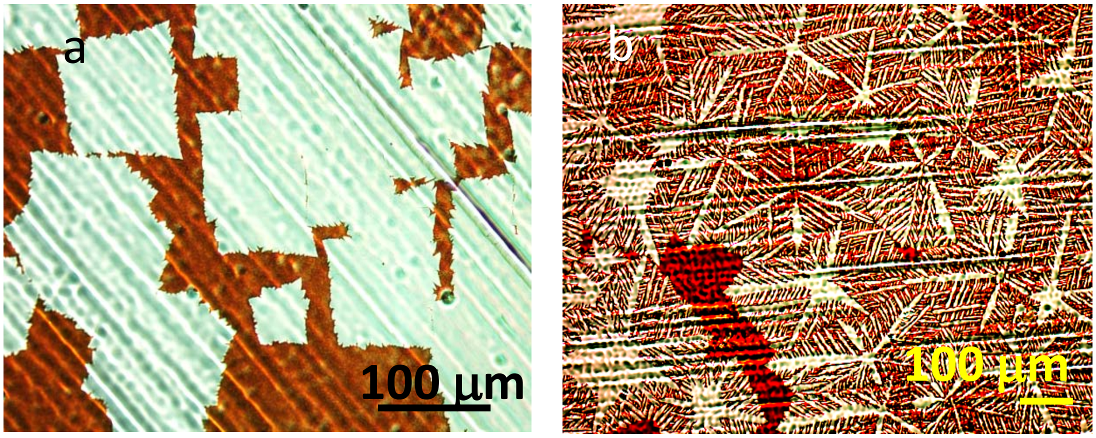

Figure S1. Typical optical microscope images of graphene (white color) on copper foil showing the effect of growth times: (a) 10 min showing unetched graphene and (b) 60 min showing etched graphene without using specific etching condition. lighter color is graphene.

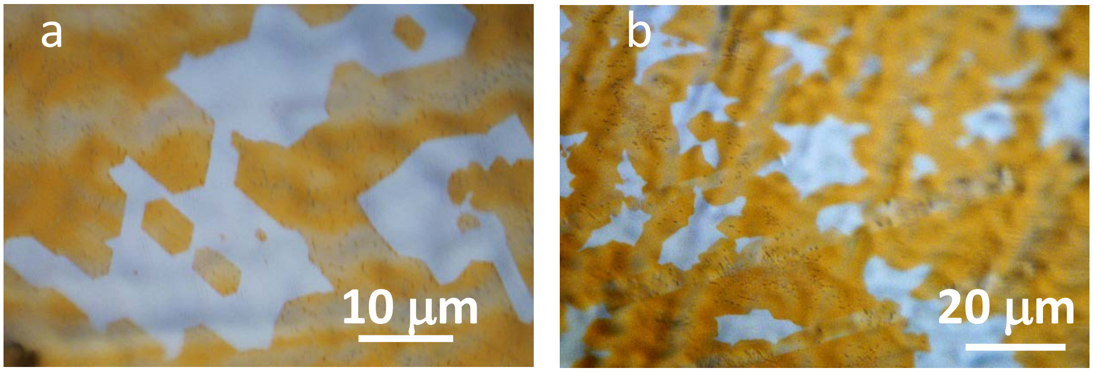

Figure S2. Optical images of oxidatively etched graphene. Graphene was first grown during 20 min with the standard protocol followed by additional 10 min growth but at reduced hydrogen flow. (a) 5 sccm H_2 showing an anisotropic etching with hexagonal holes and (b) 0 sccm H_2, the graphene is aggressively etched with the oxidative environment but still has a sign of anisotropic etching. White color is graphene.

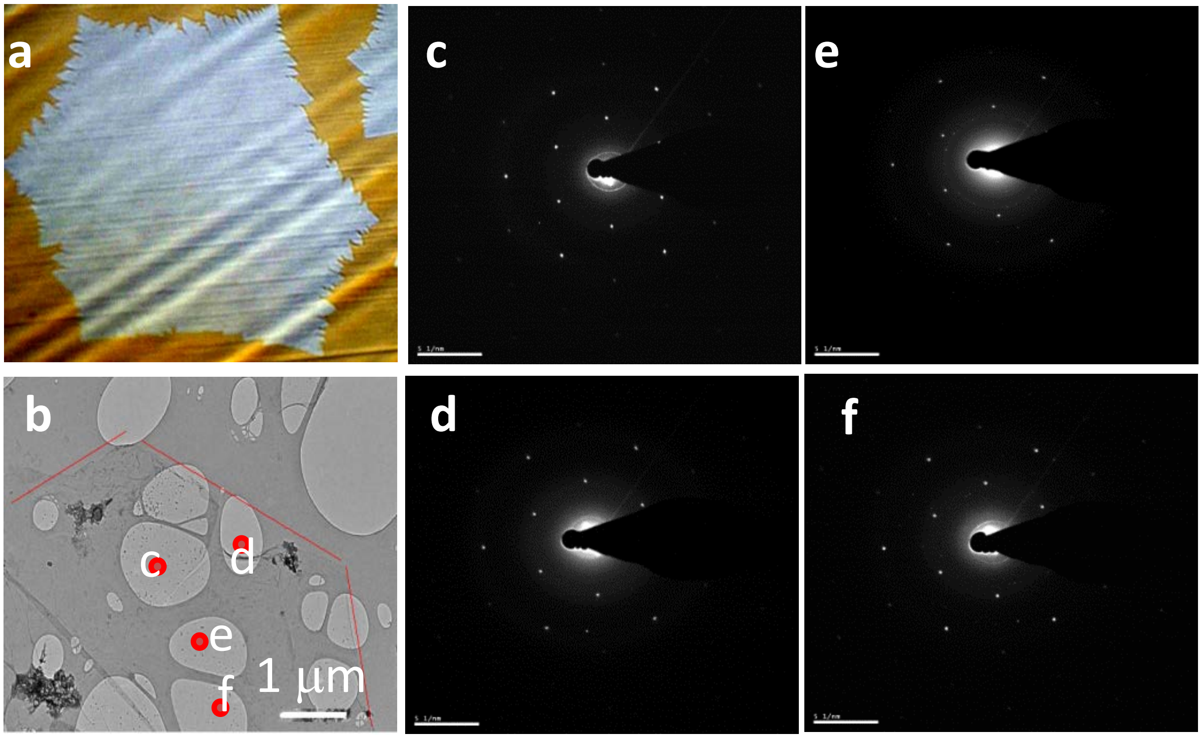

Figure S3. TEM analysis for typical graphene grown during 10 min. (a) Optical microscope image of hexagonal graphene on copper foil, (b) TEM image on TEM grid and (c-f) representative SAED patterns of single domain graphene at the four different positions indicated in (b). The electron diffraction patterns of the domain show single-crystalline nature.

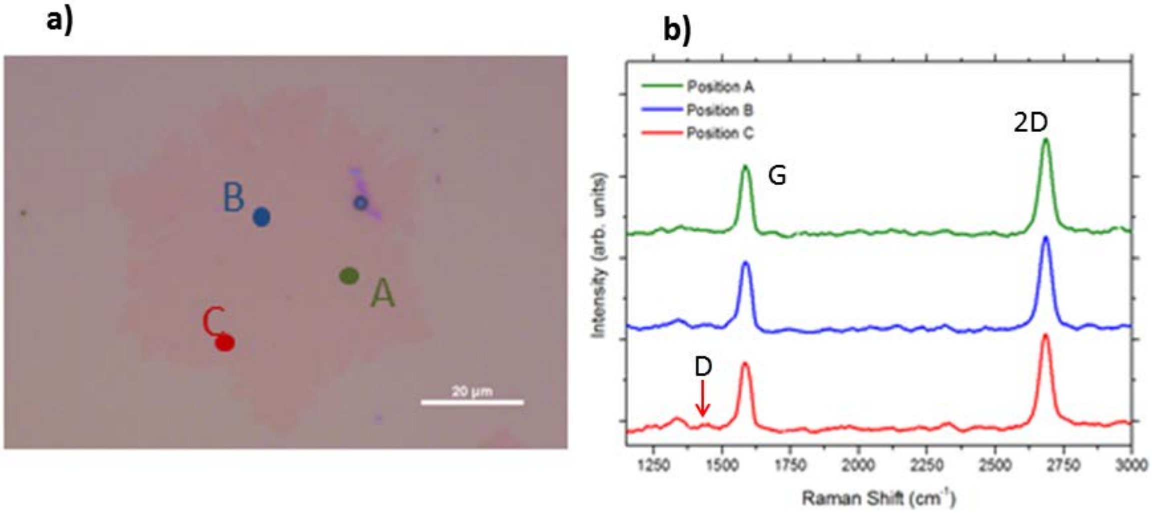

Figure S4. Raman spectra of a typical graphene flake. a) Optical image of a graphene flake grown during 20 min and transferred to SiO_2/Si (440 nm)), b) Raman spectra at 300 K for the graphene flake depicted in a) at points A,B,C showing the uniformity of the flake in the entire region. We chose three points randomly on the flake (a) and obtained the Raman G peaks at around 1585 cm^{-1} and the 2D peaks at around 2683 cm^{-1}. The presence of a small D peak below 1400 cm^{-1} could be associated with transfer related wrinkling.

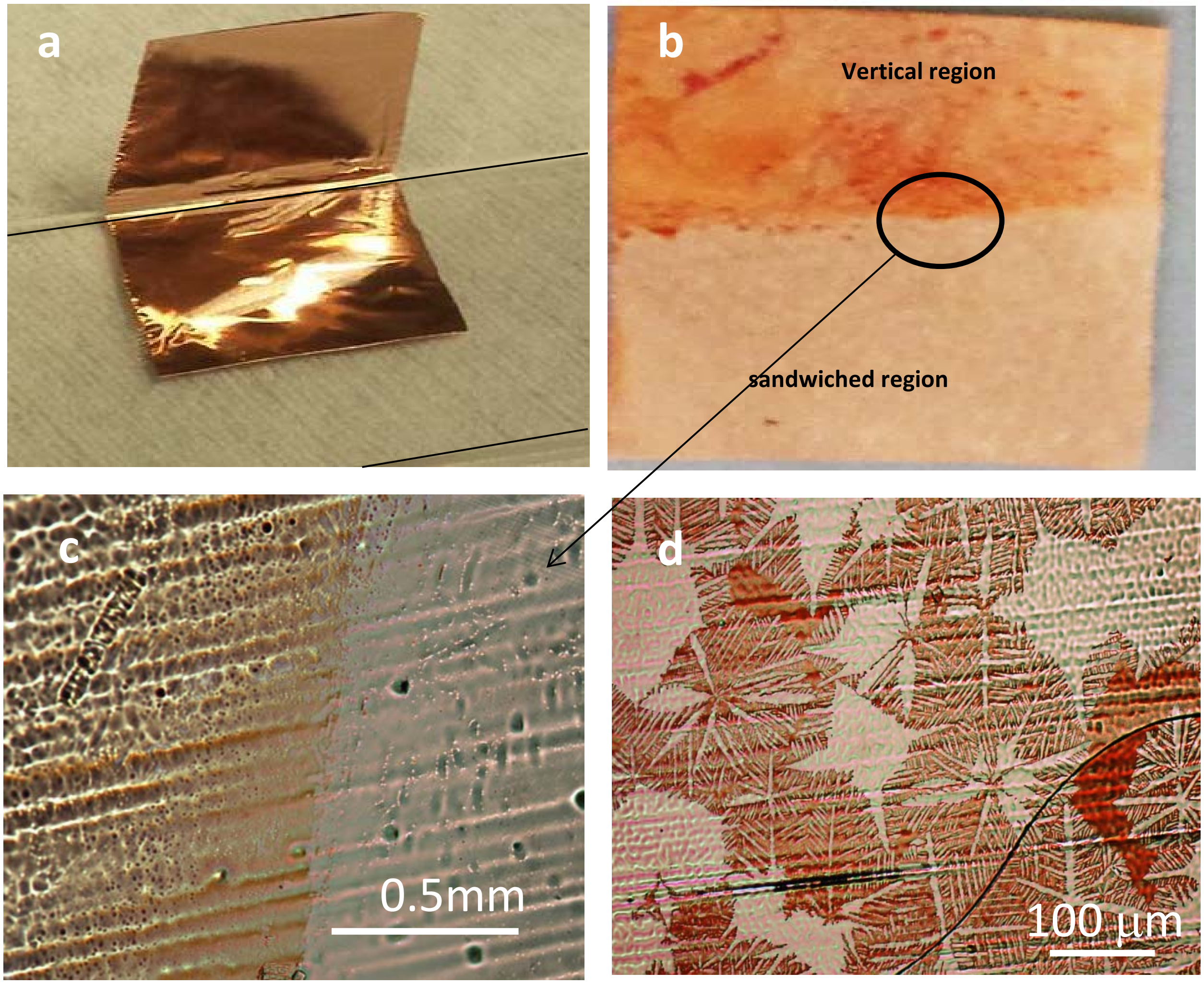

Figure S5. (a) Image of a bent copper foil ready for growth (the horizontal position is sandwiched between a quartz slide and a sample holder). (b) Photo of flattened graphene/copper foil after 60 min growth. The vertical region is reddish indicating the presence of uncovered graphene or etched region whereas the horizontal sandwiched region is whiter which indicates fully covered and unetched graphene. (c) Optical microscope image showing both vertical (left) and sandwiched (right) regions. (d) Zoomed optical image on the sandwiched region with highly etched graphene.
